# The modulatory effects of biogenic amines on male mating performance in *Bactrocera dorsalis*

**DOI:** 10.3389/fphys.2022.1000547

**Published:** 2022-09-06

**Authors:** Wenlong Chen, Yaoyao Chen, Ziwei Xiao, Yuhua Zhang, Tong Zhang, Guohua Zhong, Xin Yi

**Affiliations:** ^1^ Key Laboratory of Crop Integrated Pest Management in South China, Ministry of Agriculture, South China Agricultural University, Guangzhou, China; ^2^ Key Laboratory of Natural Pesticide and Chemical Biology, Ministry of Education, South China Agricultural University, Guangzhou, China; ^3^ Guangdong Province Key Laboratory of Microbial Signals and Disease Control, College of Plant Protection, South China Agricultural University, Guangzhou, China

**Keywords:** mating performance, Bactrocera dorsalis, biogenic amines, RNAi, pharmacological treatments

## Abstract

In insects, the emergence of mating behavior requires the interplay among sex-determination hierarchy mechanisms that regulate sex-specific differentiation, perception and integration of different sensory cues, and precisely patterned behavioral outputs. Biogenic amines, including octopamine (OA), dopamine (DA), tyramine (TA), serotonin and histamine, have been identified and proposed as putative neurotransmitters, neurohormones and/or neuromodulators in the central nervous system of insects to influence multiple physiologies and behaviors. The current study provides the physiological roles and pharmacology of these biogenic amines in the mating performance of *Bactrocera dorsalis*. Silencing gene expressions coding for biosynthetic enzymes of DA and serotonin in male flies could decrease mating rates, while OA, TA and histamine had no such effects on mating. Furthermore, injection of DA or the DA receptor antagonist chlorpromazine could affect mating rate, as well as injection of serotonin. Pharmacological treatments with other biogenic amines or their receptor antagonists in male flies have no roles in regulating mating performance. We conclude that DA and its receptors are involved in regulating male mating behaviors in *B. dorsalis*, while changes in serotonin levels in male flies could also affect mating rates. In the current study, the modulatory effects of these biogenic amines on mating performance were investigated, and these results will be helpful in providing a new strategy for controlling *B. dorsalis.*

## Introduction

Biogenic amines are widely distributed in vertebrates and invertebrates and function as neurotransmitters, neurohormones, and neuromodulators, regulating animal physiologies, and behaviors. In insects, the group of biogenic amines consists of five main members, including dopamine (DA), octopamine (OA), tyramine (TA), serotonin and histamine (Roeder, 2005; Pirri et al*.*, 2009; Blenau and Thamm, 2011; Farooqui, 2015; Verlinden, 2018). Among them, DA, serotonin and histamine are found in both vertebrates and invertebrates, while OA and TA are specific to invertebrates. The counterparts of OA and TA in vertebrates are epinephrine and norepinephrine, which share similar structures and functions. These biogenic amines are widely distributed in the central nervous system (CNS) of insects and can modify neural functions at multiple levels (Evans, 1980; Mercer et al*.*, 1983; Monastirioti, 2003; Thomas et al*.*, 2003; Scheiner et al*.*, 2006). They can bind to specific receptors and act on specific sensory organs or particular groups of muscle fibers, thus further affecting the sensation and movement of insects (Horner, 1999; Scheiner et al*.*, 2002). In addition, biogenic amines also play key roles in modulating stereotyped behaviors, including associative learning and memory formation (Hammer and Menzel, 1998; Kononenko et al*.*, 2009; Riemensperger et al*.*, 2011; Fukumitsu et al*.*, 2012). The function of biogenic amines varies considerably in insects, thus, a detailed investigation in specific species is necessary.

Courtship and mating behaviors are fundamental to the propagation of insect populations. Upon insect mating, they use multiple sensory cues to recognize conspecifics and to choose a suitable partner to mate for reproduction (Bradbury and Vehrencamp, 2012). A well-established insect model for the study of courtship and mating behavior is *Drosophila melanogaster*. During the courtship phase, fruit flies rely on the exchange of auditory, visual, and chemosensory signals, and some of them can stimulate male or female courtship, while others can inhibit courtship (Everaerts, 2006). Thus, the occurrence of courtship behavior is closely associated with sensory sensitivities, which could be regulated by biological amines. Previous studies demonstrated that downregulating DA levels in male flies increased their attractiveness to other males, as changes in DA levels affect the perception of some sensory signals in male flies (Liu et al*.*, 2008; Liu et al*.*, 2009). In addition, biogenic amines regulate the processes of pheromone synthesis. Previous studies demonstrated that OA moderated the synthesis processes of sex pheromones in both *Helicoverpa virescens* and *Helicoverpa zea*, as well as DA in female *D. melanogaster* (Christensen et al*.*, 1991; Christensen et al*.*, 1992; Abrieux et al*.*, 2013). Therefore, biogenic amines play important roles in regulating courtship and mating behavior in insects.

The oriental fruit fly, *Bactrocera dorsalis* (Diptera: Tephretidae) is a global highly-invasive pest in agricultural production that possesses high dispersal capacity and broad climate tolerance. This species causes a substantial loss of cultivated crops throughout most of Asia and damages more than 250 host fruits and vegetables worldwide (Zeng et al*.*, 2018; Liu et al*.*, 2019). Previous studies have shown that biogenic amines play important roles in pupation, immune regulation, olfactory learning, and gut bacterial community homeostasis in *B. dor*s*alis* (Chen et al., 2018; Yu et al., 2022; Zeng et al., 2022). However, their roles in regulation of courtship and mating behavior in *B. dor*s*alis* remain elusive. Courtship behavior is one of the most complex behaviors in insects, involving the interplay of multiple sensory modalities between males and females, and the plasticity of mating behaviors under different circumstances relies on integrating pheromone perception with internal state information and external stimuli to synergistically regulate mating behaviors. Due to their abilities to act on all levels of insect nervous system, biogenic amines could serve as downstream key mediators to trigger adjustments to courtship behaviors and pheromone sensings in response to the plasticity of mating. However, the behavioral roles of biogenic amines are wide-ranging in different forms of behaviors and in different insects. In this study, molecular and pharmacological methods were applied to examine the effects of biogenic amines on the mating performance of male *B. dor*s*alis*, and it was found that DA and its receptors were involved in regulating male mating behaviors, while changes in serotonin levels in male flies could also affect mating rates*.* Illuminating the regulatory roles of biogenic amines in mating behaviors could provide insights into the control strategy against *B. dorsalis* by inhibiting mating behaviors and provide an overview of the functional roles of biogenic amines in mating behaviors of *B. dorsalis.*

## Materials and methods

### Insect rearing

*B. dorsalis* was obtained from a laboratory-reared colony (Key Laboratory of Pesticide and Chemical Biology, South China Agricultural University, Guangzhou, China). They were collected from citrus plants (*Citrus reticulata* Blanco) in Guangzhou, Guangdong Province, in 2010, and were maintained in an artificial climate chamber at 27 ± 0.5°C, 60%–80% relative humidity, and a 14:10 h photoperiod.

Adult flies were reared on artificial diets consisting of yeast extract (25%) and sugar (75%). Larval diets consist of banana (31.8%), corn (31.8%), yeast extract (3.2%), sugar (3.2%), sodium benzoate (0.1%), fiber (paper towel, 3.2%), hydrochloric acid (0.2%) and distilled water (26.5%) (Gu et al., 2022).

To prevent mating, we separated male and female flies on the third day post emergence and placed male and female flies into separate chambers (Markow, 1985). Each chamber (30 cm × 30 cm × 30 cm) consisted of 500 same-sex flies, and those males without contact with females since the third day post emergence are considered “virginal ♂” (Yu et al., 2022). Males that have mated only once with females since emergence are considered as “mated ♂“, and were used for subsequent experiments within 24 h post mating. On the 13th day post emergence, to observe their mating processes, 10 females and 10 males were randomly selected and put together into a cylindrical transparent box (diameter: 10 cm; height: 25 cm) with a constant diet supply, and the mating process was monitored as previously indicated (Lone et al., 2015). When we observed copulation between male and female lasting for at least 10 min, this couple was considered as successful mating (Bougleux Gomes et al., 2019). Each mating trial consisted of 10 parallel courtship matches (10 males and 10 females), and the independent mating trial was repeated four to eight times. The mating rate was defined as the percentage of fly couples engaged in successful mating during the 1-h observation. Mating latency was measured as the time taken from introducing flies into the chamber till the initiation of copulation (Toda et al., 2012; Rose et al., 2022). All the mating trials were performed at 8:00 p.m., which falls in the mating peak of *B. dorsalis* (Norio et al., 1984). All flies were tested only once.

### Quantitative real-time PCR examinations

TRIzol (AG21102, AG, China) was used to extract the total RNAs from male *B. dorsalis* under different states*.* The total RNA was determined by 1.2% agarose gel electrophoresis. The concentrations and purity of the total RNAs were determined by a NanoDrop 2000 spectrophotometer (Thermo Fisher Scientific, United States ). The cDNA template was synthesized according to the instruction manual of the *Evo M-MLV* RT Kit (AG11711, AG, China), and the reverse transcription products were stored at −20°C. The specific primers used in RT-qPCR analysis are listed in Supplementary Table S1. RT-qPCR analysis was performed on a CFX96 Touch Real-time PCR Detection System (Bio-Rad, Hercules, CA) using the SYBR Premix Ex Taq II kit (Takara, China). The reference gene *EF1a* was used to normalize the expression level (Wang et al., 2010). For each mating trial, RNA of male flies from successful mating couples was extracted and considered as one biological replicate. For statistical analysis, RNAs of male flies from other independent mating trials were also extracted and subjected to RT-qPCR analysis. During RT-qPCR analysis, three technical replicates were set up for every sample to ensure the accuracy of the data and sample stability. Those isolated male flies without contact with females under the same developmental stages (13th day post emergence) were served as the control.

### RNA interference treatment

The full-length cDNA sequences of biosynthetic enzymes of biogenic amines in *B. dorsalis* were obtained from National Center for Biotechnology Information (NCBI) (Supplementary Datasheet S1), and specific primers with the T7 promoter sequence at the 5′ end were designed (Supplementary Table S1 **)**. Total RNA was extracted as a template, and fragments of five genes, *tyrosine hydroxylase* (*Th*), *tyrosine-β-hydroxylase* (*Tβh*), *tryptophan hydroxylase* (*Tph*), *tyrosine decarboxylase* (*Tdc*), and *histidine decarboxylase* (*Hdc*), were amplified by PCR. A universal DNA Purification Kit (TIANGEN, China) was used for purification and then linked to PMD-18T (TaKaRa, China) for transformation and sequencing. After sequencing, a TIANprep Mini Plasmid Kit (TIANGE, China) was used to extract plasmid and enzyme digestion, and the enzyme digestion product was used to synthesize dsRNA with a T7 RiboMAX Express RNAi System (P1700, Promega, United States). Simultaneously, green fluorescent protein (GFP) was used as a control. The synthesized dsRNAs were purified using an RNeasy MinElute Cleanup Kit (Qiagen, Germany). The size of the dsRNA product was confirmed by electrophoresis on a 1.5% agarose gel, and the final concentration of dsRNA was adjusted to 3.5 μg/μl. Candidate flies (10th day post emergence) were fixed on slides after anaesthetization by carbon dioxide, and 1 μg of dsRNA was injected into the abdomen of male files by a microloader (Eppendorf, Hamburg, Germany). Control flies at the same stage were injected with equivalent volumes (1 μg) of ds*GFP*. One hundred flies were injected for each treatment. After injection, all files were then reared under standard rearing conditions (27 ± 0.5°C with 60%–80% relative humidity in a 14 : 10 h photoperiod), and the expression levels were examined at 72 h after injection by qPCR. After the confirmation of gene knockdown, male flies (13th day post emergence) injected with dsRNAs (or control group that received ds*GFP*) were then placed together with untreated female flies (13th day post emergence), and the mating behaviors were monitored as aforementioned.

### Pharmacological assays

3-Hydroxytyramine hydrochloride, octopamine hydrochloride, tyramine hydrochloride, serotonin hydrochloride, chlorpromazine hydrochloride, epinastine hydrochloride, and yohimbine hydrochloride were purchased from Macklin (Shanghai, China) and dissolved in RNase-Free Deionized water (DEPC water) at concentrations of 0.5 μg/μl and 0.05 μg/μl, respectively (concentration selection was followed by previous study) (Lenschow et al., 2018; Li et al., 2020; Yu et al., 2022). Male flies (13th day post emergence) were injected with 0.4 µL of chemicals at a concentration of 0.5 μg/μl or 0.05 μg/μl, respectively. Equal amounts of DEPC water were injected into male flies and served as a control. Two h post injection, 10 untreated virgin females (13th day post emergence) and 10 injected males were put into cylindrical transparent boxes for monitoring mating behaviors as aforementioned.

### Statistical analyses

All data obtained in these experiments were statistically analyzed and plotted by GraphPad Prism 8.0 and SPSS Statistics 26.0. Log-rank (Mantel-Cox) test (Toda et al., 2012) and Students’s *t* test were used to analyze statistical difference in mating latency and mating rate, respectively. The analysis results were expressed as *p* values. All the data obtained by RT-qPCR were analyzed by the 2^*−△△C*^_*t*_ method (Wang et al., 2010).

## Results

### Gene variations under different mating states

**FIGURE 1:**
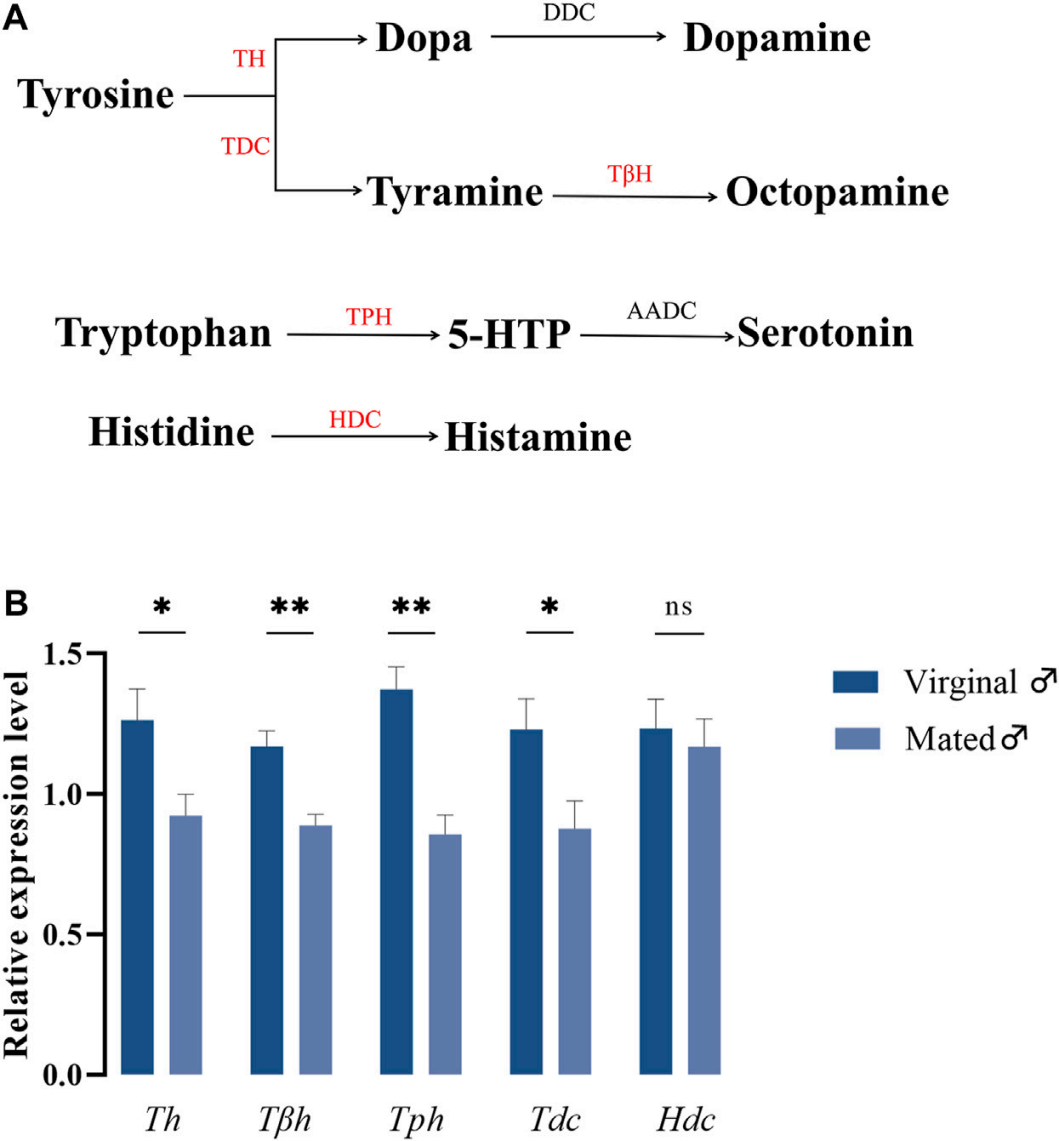
The roles of biogenic amines in *B. dorsalis*. **(A)** Synthesis pathways of biogenic amines in insects. The candidate enzymes were represented in red font. TH, tyrosine hydroxylase; DDC, dopa decarboxylase; TDC, tyrosine decarboxylase; TβH, tyrosine-β-hydroxylase; TPH, tryptophan hydroxylase; AADC, 5-hydroxytryptophan decarboxylase; HDC, histidine decarboxylase. **(B)** The variations of genes coding for biosynthetic enzymes under different mating states. Values are the means (±SEs) of replicates. Statistical comparisons were based on Students’s *t* test. The level of significance for the results was set at *p < 0.05, **p < 0.01.

In insects, it has been widely accepted that biogenic amines are closely associated with their respective biosynthetic enzymes, and the biosynthetic pathways for these five amines are presented in Figure 1A. To understand whether different mating states would lead to variations in gene expressions coding for biosynthetic enzymes of biogenic amines, RT-qPCR was performed to detect the relative expression changes of these five genes in virginal and mated males (13th day post emergence). The results indicated that mating could significantly downregulate the expressions of many amine-synthesis genes, except for *Hdc*, which is responsible for the synthesis of histamine (Figure 1B)*.*

### Silencing genes coding for biosynthetic enzymes of biogenic amines could influence the mating behaviors of male flies

**FIGURE 2:**
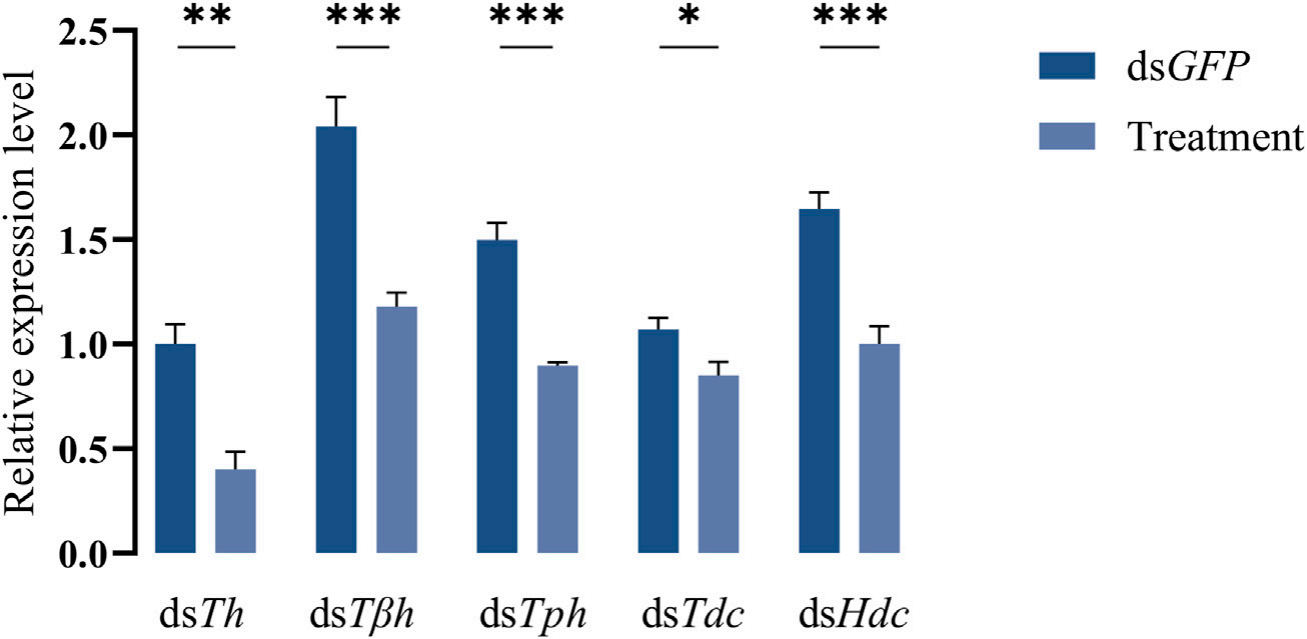
Efficiency of RNAi. The flies injected with ds*GFP* were served as control, and treatments represented the flies receiving dsRNA targeting different genes. Values are the means (±SEs) of replicates. Statistical comparisons were based on Students’s *t* test. The level of significance for the results was set at *p < 0.05, **p < 0.01, ***p < 0.001.

**FIGURE 3:**
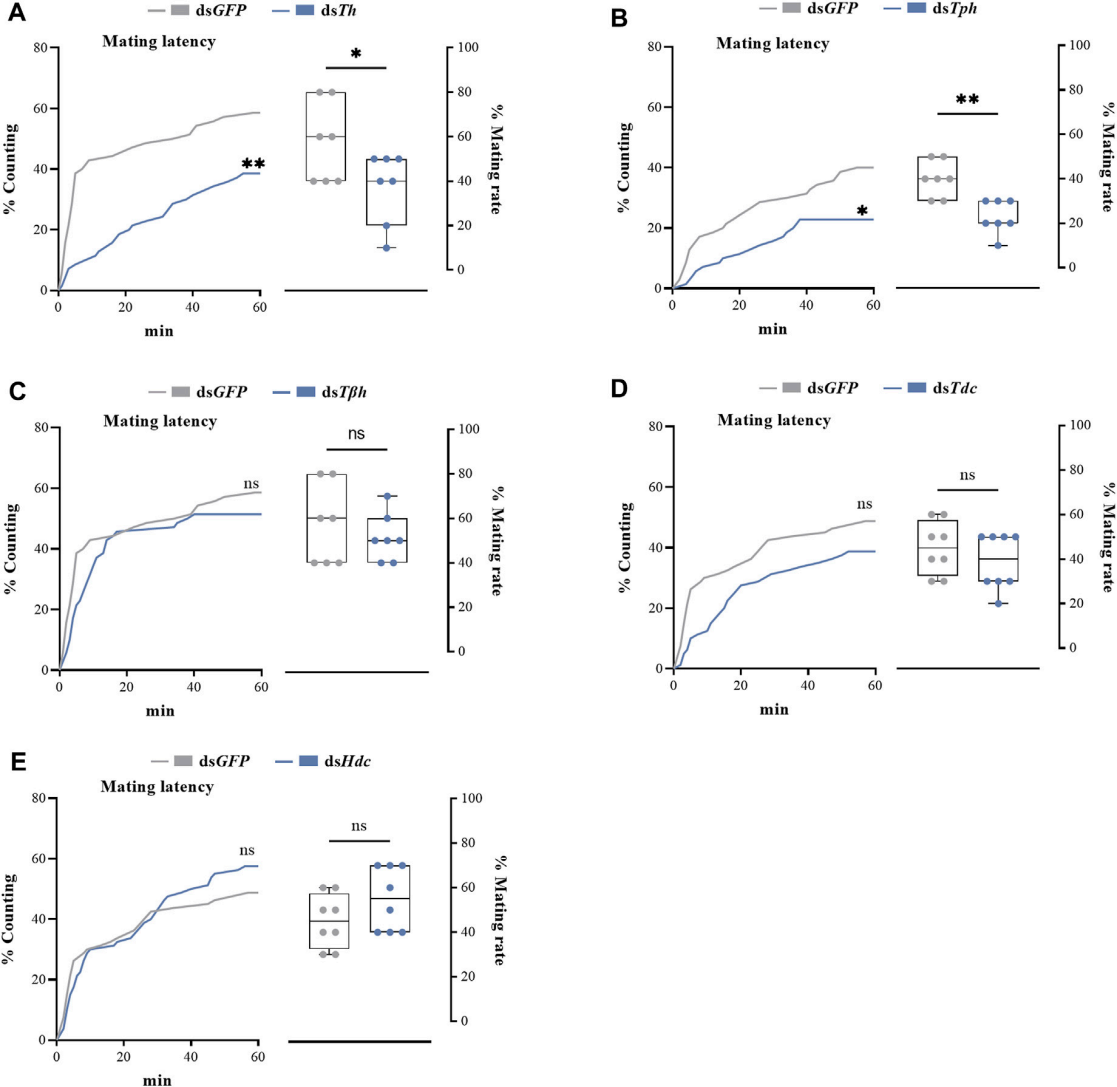
The roles of genes coding for biosynthetic enzymes of biogenic amines, including *Th* **(A)**, *Tph* **(B)**, *Tβh* **(C)**, *Tdc* **(D)** and *Hdc* **(E)**, in mating latency (left) and mating rate (right) of *B. dorsalis*. Mating assays were performed at 72 h after the injection of dsRNAs in virginal male flies (13th day post emergence). Values are the means (± SEs) of replicates. Statistical comparisons were based on Log-rank (Mantel-Cox) test (mating latency) and Students’s *t* test (mating rate). The level of significance for the results was set at *p < 0.05, **p < 0.01, ***p < 0.001.

dsRNAs targeting different genes coding for biosynthetic enzymes of biogenic amines were injected into the abdomen of virginal male flies (10 days post-emergence). The relative expression levels of target genes were detected by RT-qPCR at 72 h post dsRNA injection. Compared with ds*GFP* treatment, the relative expression levels of all candidate genes in flies injected with dsRNAs were significantly decreased (Figure 2). We then recorded the mating rates of these RNAi-treated male flies with untreated female flies. The results indicated that compared with the control group (flies receiving ds*GFP*), the mating rates of males injected with ds*Th* and ds*Tph* were significantly decreased (Figures 3A,B), indicating that DA and serotonin might participate in regulating male mating behaviors in *B. dorsalis*. In contrast, there were no significant changes in the mating rates after male flies were injected with ds*Tβh*, ds*Tdc* or ds*Hdc* compared with those of *dsGFP* treatments (Figures 3C–E)*.* These results indicated that silencing gene expressions of *Th* and *Tph* could significantly affect male mating performance, but *Tβh*, *Tdc* and *Hdc* seemed to have no roles in regulating male mating behaviors in *B. dorsalis.* The time spent by the two potential mates till they are ready to go for the copulation is termed as mating latency (Pavkovi-Luci and Keki, 2009). And mating latency is recognized to be an important component of mating behavior. We then also examined the mating latency following dsRNA treatments, and found that only flies injected with *dsTh* or *dsTph* had lower mating latency (Figures 3A,B), which indicated that flies with lower levels of DA or serotonin were slower to initiate courtship and might have weaker desire to mate compared with *dsGFP* treated control flies.

### Effects of biogenic amine injection on the mating behaviors of male flies

**FIGURE 4:**
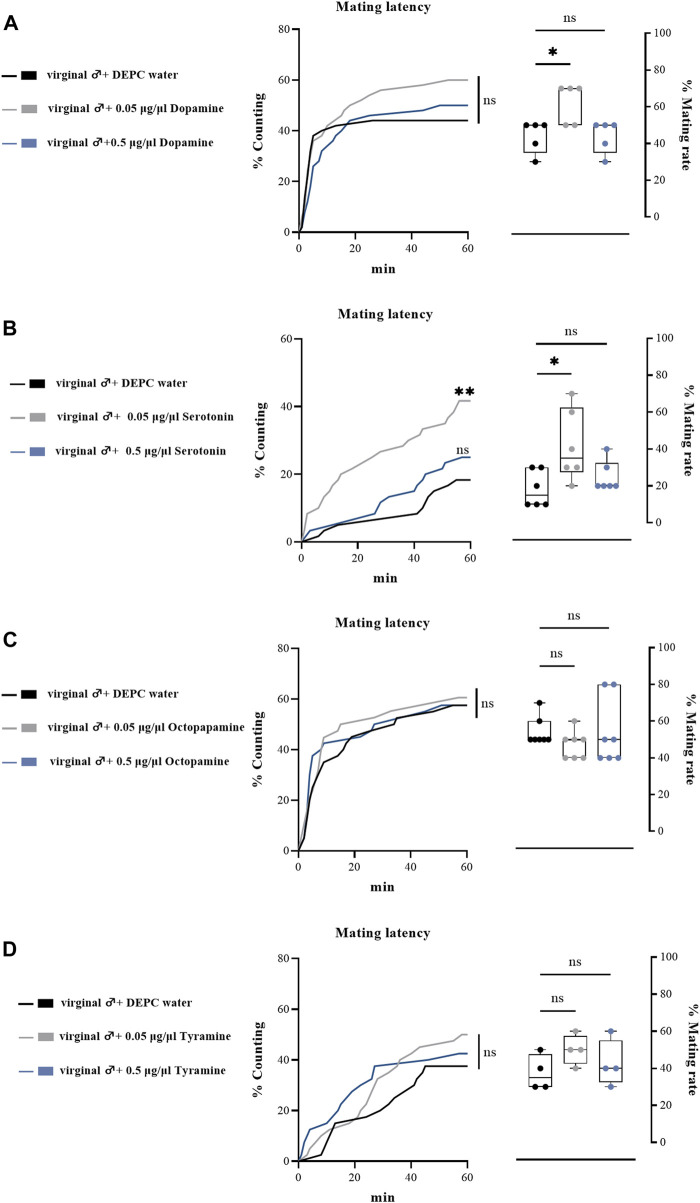
Changes in mating latency and mating rates following pharmacological treatments. Mating assays were performed at 2 h after biogenic amine-injection in virginal male flies (13th day post emergence). DEPC water treatment was served as control. For treatment groups, 0.05 μg/μl dopamine **(A)**, serotonin **(B)** and octopapamine **(C)** and tyramine **(D)** were injected, respectively. Values are the means (± SEs) of replicates. Statistical comparisons were conducted by comparing each treatment with control, respectively, and based on Log-rank (Mantel-Cox) test (mating latency) and Students’s *t* test (mating rate). The level of significance for the results was set at *p < 0.05, **p < 0.01, ***p < 0.001.

**FIGURE 5:**
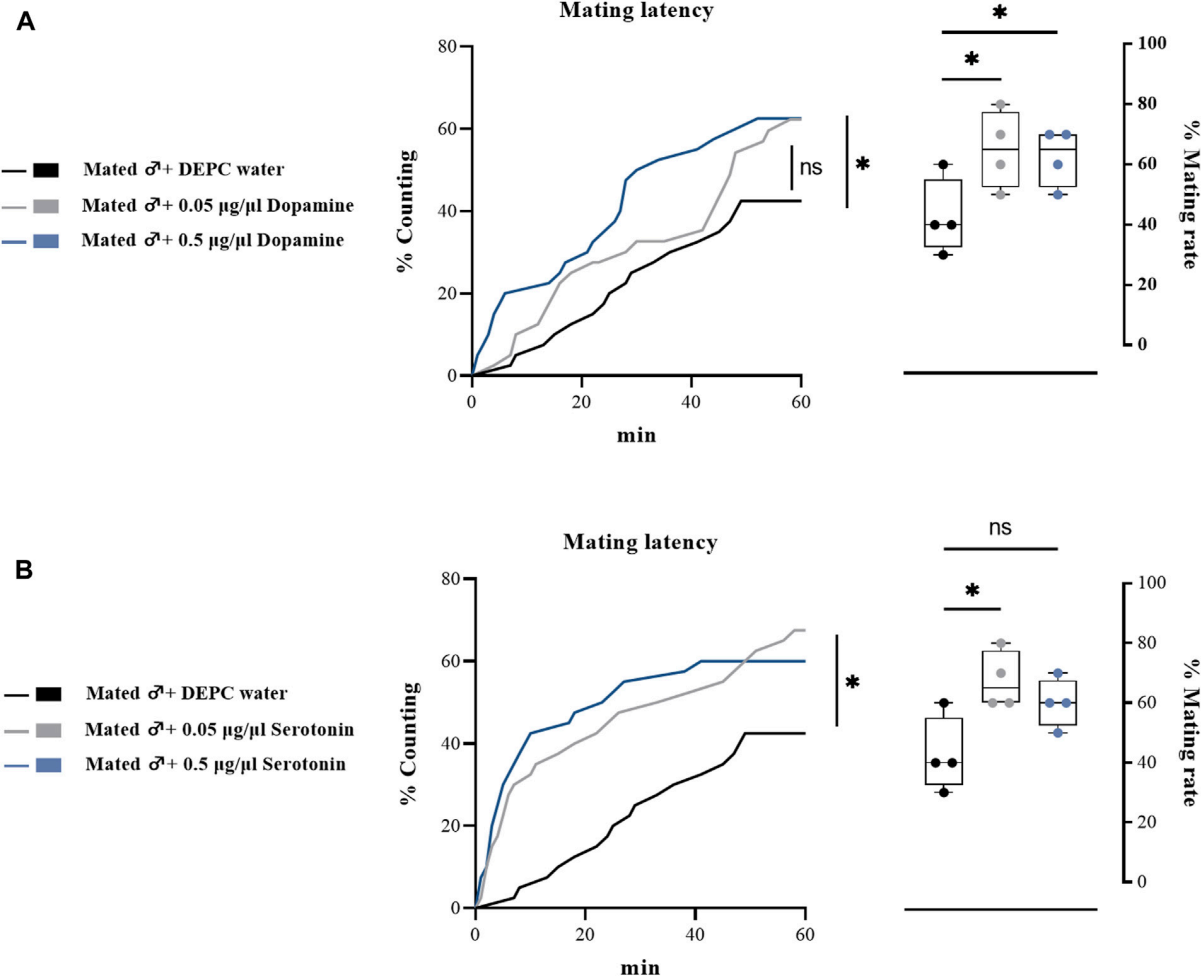
Changes in mating latency and mating rates following pharmacological treatments in mated male flies. Mating assays were performed at 2 h after biogenic amine-injection in mated male flies (13th day post emergence). DEPC water treatment was served as control. For treatment groups, 0.05 μg/μl dopamine **(A)**, and serotonin **(B)** were injected, respectively. Values are the means (± SEs) of replicates. Statistical comparisons were conducted by comparing each treatment with control, respectively, and based on Log-rank (Mantel-Cox) test (mating latency) and Students’s *t* test (mating rate). The level of significance for the results was set at *p < 0.05, **p < 0.01, ***p < 0.001.

To further determine the effects of biogenic amines in regulating the mating behaviors of *B. dorsalis*, we injected different types of biogenic amines into virginal male flies (13th day post emergence). We found that 0.05 μg/μl DA injection into virginal male flies significantly upregulated the mating rate compared with control flies (DEPC water injected flies), but such increasing was abrogated by a higher concentration of DA (0.5 μg/μl) injection (Figure 4A). However, the mating latency was not significantly influenced following DA-injection (Figure 4A). As well as the situation for serotonin, only a low concentration of serotonin (0.5 μg/μl) increased the mating rate and mating latency (Figure 4B). Moreover, OA and TA injection had no influences on the mating performance of *B. dorsalis* (Figures 4C,D). These results could nicely match the results from RNAi, which suggested that only DA and serotonin might have roles in regulating male mating behaviors in *B. dorsalis*. We then continued to compare the mating performance by injection DA and serotonin into mated flies (13th day post emergence). The results showed that injection low concentration of DA and serotonin (0.05 μg/μl) into mated flies could also enhance mating by increasing mating rate and mating latency (Figure 5).

### Effects of receptor antagonist injection on the mating behaviors of male flies

**FIGURE 6:**
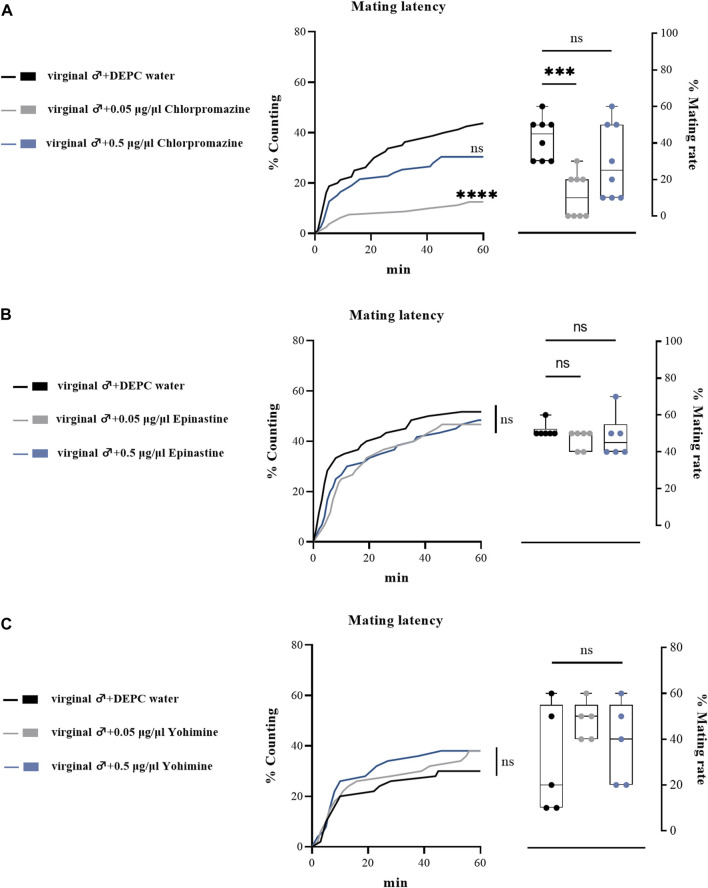
Changes in mating latency and mating rates following injection of biogenic amines receptor antagonists in virginal male flies. Mating assays were performed at 2 h after injection on virginal male flies (13th day post emergence). DEPC water treatment was served as control. For treatment groups, 0.05 μg/μl chlorpromazine **(A)**, epinastine **(B)** and yohimine (C) were injected, respectively. Values are the means (± SEs) of replicates. Statistical comparisons were conducted by comparing each treatment with control, respectively, and based on Log-rank (Mantel-Cox) test (mating latency) and Students’s *t* test (mating rate). The level of significance for the results was set at *p < 0.05, **p < 0.01, ***p < 0.001.

We also performed a subset of experiments by injecting biogenic amine receptor antagonists into virginal male flies to examine their effects on mating performance. Consistent with previous results, only a relative low concentration of DA receptor antagonist (chlorpromazine) injection influenced male mating behavior, as only 0.05 μg/μl chlorpromazine hydrochloride injection significantly downregulated the mating rate and mating latency (Figure 6A), while epinastine hydrochloride (OA receptor antagonist) and yohimbine hydrochloride (TA receptor antagonist) injection led to no changes in mating rates and mating latency compared with the control groups (Figures 6B,C). In line with the abovementioned results, we suggested that only a low concentration of chlorpromazine hydrochloride could have effects on the mating behaviors of *B. dorsalis.*

## Discussion

The results from this study could be an important step in unraveling the neuromodulatory mechanism underlying mating behaviors in *B. dorsalis*. Accurate sensing by insects is crucial for mating, and neuromodulators interacting at different levels of the olfactory system could trigger variability in the behavioral responses of insects to sex pheromones, thereby regulating mating behaviors (Liu et al., 2009). DA is an important integrator between central nervous system and mating behavior outputs in insects. A previous study indicated that males with increased DA levels induced by a pharmacological approach showed an enhanced propensity to court other males but did not change their courtship toward virgin females, general olfactory response, general gustatory response or locomotor activity (Liu et al., 2008). Female *Nasonia vitripennis* fed a DA-receptor antagonist prior to mating maintained their attraction to male pheromone after mating, while virgin females injected with DA became unresponsive (Lenschow et al., 2018). Therefore, female and male insects might elicit different reactions in response to changes in DA levels. Our results indicated that injection of a relatively low concentration of DA into male *B. dorsalis* could significantly promote mating with females, while injection of a DA receptor antagonist (chlorpromazine) or knockdown of the gene encoding the biosynthetic enzyme of DA (*tyrosine hydroxylase*) could partially impair the desire for mating. The DA signal is transmitted through the D1-like DopR2 receptor to P1 neurons, which also integrate sensory information relevant to the perception of females and project to courtship motor centers that initiate and maintain courtship behaviors (Clowney et al., 2015; Zhang et al., 2016; Zhang et al., 2018). As in a previous study, we proposed that depleted DA induced distraction of males during mating, difficulties in properly orienting toward the female, attention of males toward inappropriate female body parts, and thus increased latency to orientation might contribute to worse mating performance (Neckameyer, 1998). Moreover, flies with reduced level of DA decreased mating desire which resulted in them reacting slowly. According to Markow (1978), males with high vigor react quickly in the presence of a female whereas males with less mating vigor react slowly. Thus, changes in DA levels in male insects would be sufficient to calibrate mating behaviors by DA neurons of the brain, and dopaminergic neurons located in the ventral nerve cord could further determine the persistence and duration of mating. In addition, studies have shown that altered DA levels have a “U” shaped effect on courtship of *Drosophila* males, with either high or low DA levels promoting courtship behavior in males flies (Liu et al., 2008; Liu et al., 2009). And excess dopamine and serotonin have no effect on the mating receptivity of insect females (Yamane and Miyatake, 2010). The mechanism underlying different concentrations of DA or DA receptor antagonists leading to different reactions needs further investigation.

In the current study, another biogenic amine with effects on mating behaviors was serotonin. Serotonin is a monoamine neurotransmitter that is expressed within discrete circuits in the brain and ventral nerve cord and is essential for normal courtship and mating (Hull et al., 2004). Similar to the role of DA in male flies, we found that injection of serotonin could significantly increase the mating rate, and silencing the expression of a biosynthesis enzyme (*tryptophan hydroxylase*) suppressed mating in *B. dorsalis*. Likewise, injecting an antagonist of the Bm5-HT1A receptor or dsRNA of *Bm5-HT1A* into *Bombyx mori* caused lowered courtship vitality or moving speed (Xiong et al., 2019). Treatment with the serotonin receptor antagonist SB25871 or knockdown of the *5-HT*_*7*_ *Dro* gene interfered with the courtship and mating behaviors of male flies by disrupting intermediate behaviors (licking, curing, and attempts) in *Drosophila* (Becnel et al., 2011). Thus, we suggested that the decline in mating activity by silencing *tryptophan hydroxylase* in *B. dorsalis* might be attributed to the drop in motion abilities. It has been shown that serotonin also enhances the sensitivities of antennal lobe neurons for sex pheromones in *Manduca sexta* by increasing the excitability of central olfactory neurons (Kloppenburg et al., 1999). However, Brent et al. (2016) found that artificial injection of serotonin into females did not affect their sexual acceptance capacity of *Lygus hesperus* Knight (Hemiptera: Miridae). Such different reactions and mating behaviors in males and females triggered by changes in serotonin levels might imply sexual dimorphism, which needs further investigation.

OA is involved in a series of important behaviors and physiological activities, including olfactory learning and memory, rhythmic behaviors, aggression, and energy metabolism (Sombati and Hoyle, 1984; Hammer and Menzel, 1998; Mentel et al., 2003). TA, which is regarded as the precursor of OA, has neural functions in insects (Lange, 2009). OA and TA were found to be essential in the regulation of oviposition through female OA neuronal signaling (Li et al., 2020). *Tβh* is the enzyme that converts TA to OA. Genetic studies of *Drosophila* revealed that *Tβh* mutation produced female sterility due to defective egg-laying (Monastirioti, 2003). In this study, the expression levels of *Tβh* and *Tdc* were relatively lower in mated male flies than in virginal flies. Previous studies indicated that males with no OA or with low OA levels do not adapt to changing sensory cues and court both males and females by integrating multiple sensory modalities (Certel et al., 2007). Moreover, deletion of the gene coding for *Tβh* decreased aggression in flies but without affecting courtship (Zhou et al., 2008). In addition, flies exhibited more intense male-male courtship after knocking down *Tdc2* ^*RO54*^, the enzyme gene required for TA synthesis (Huang et al., 2016). These results were in agreement with our results, which showed that silencing genes coding for biosynthetic enzymes of TA and OA have no significant roles in regulating male-female mating behaviors in *B. dorsalis.* These findings indicated that OA and TA might not have direct effects on male-female mating behavior. Previous studies have shown that TA and OA could be involved in regulating male-male mating behavior in *Drosophila*, but treated males continued to mate normally with females when females were present (Zhou et al., 2008; Huang et al., 2016). Although our data suggest that OA and TA do not have direct effects on female-male mating behaviors, it cannot be excluded that they are involved in regulating male-male mating processes. Therefore, the specific functions of OA and TA in the mating behaviors of *B. dorsalis* need to be further investigated.

Previous studies found that neuronal histamine can act as an alarm signal in the brain for activities such as exploration, self-defense and stress response and plays an important role in sleep-wake, thermoregulation and feeding (Hong et al., 2006; Thurmond et al., 2008; Yamada et al., 2020; Aryal and Lee, 2021). As a photoreceptor transmitter, histamine acts on ligand-chloride channels. In addition to its presence in photoreceptor cells, histamine is distributed in a rather small number of neurons in the insect brain (Pollack and Hofbauer, 1991; Borycz et al., 2000; Witte et al., 2002). We downregulated histamine levels by silencing *Hdc* and found that such a decrease did not affect the mating behaviors of *B. dorsalis*. There are few reports underlying the role of histamine in courtship and mating behavior in invertebrates. Only Par et al. (2003) found that knocking down the expression of *Hdc* in mice significantly inhibited the mating behavior but did not affect their mating success. These results suggested that histamine might be involved in regulating but not indispensable for mating behaviors in mammals, however, its role in insects needs to be further determined.

## Conclusion

Successful mating in insects depends on multiple physiological and behavioral processes that take place in a timely and orderly manner in both mating partners. In addition, mating processes need to be well adjusted and coordinated by both external and internal factors. In this study, we found that silencing genes coding for biosynthetic enzymes or pharmacological treatments of DA and serotonin in males could significantly influence the mating behaviors of *B. dorsalis*, implying their roles in regulating male mating behaviors. However, with the HPLC-MS method, we failed to establish detection methods to examine the levels of biogenic amines. In future studies, it is essential to monitor the changes in biogenic amines following different treatments. These results identified the potential roles of these neuromodulators in the functioning related to mating behaviors, but the detailed regulatory mechanisms need to be further elucidated.

## Data Availability

The raw data supporting the conclusions of this article will be made available by the authors, without undue reservation.
