# Supplementary material for: The modulatory effects of biogenic amines on male mating performance in Bactrocera dorsalis
Source: Front Physiol. 2022 Sep 6;13:1000547. doi: 10.3389/fphys.2022.1000547 (PMC9486026; doi:10.3389/fphys.2022.1000547)
Supplement: Supplementary file 1 [file Table1.DOCX]

**The modulatory effects of biogenic amines on male mating performance in *Bactrocera dorsalis***

**Wenlong Chen^1,2#^, Yaoyao Chen^1,2#^, Ziwei Xiao^1,2^,Yuhua Zhang^1,2^, Tong Zhang^3^, Guohua Zhong^1,2^^[[1]](#footnote-0)^* and Xin Yi^1,2^^[[2]](#footnote-1)^***

^1^ Key Laboratory of Crop Integrated Pest Management in South China, Ministry of Agriculture, South China Agricultural University, Guangzhou, China

^2^ Key Laboratory of Natural Pesticide and Chemical Biology, Ministry of Education, South China Agricultural University, Guangzhou, China

^3^ Guangdong Province Key Laboratory of Microbial Signals and Disease Control, College of Plant Protection, South China Agricultural University, Guangzhou, China

**Supplementary Table 1**  Primers used for RT-qPCR and double stranded RNA synthesis.

| Primer name | Forward primer sequence | Primer name | Reverse primer sequence |
| --- | --- | --- | --- |
| *EF1a*-F | CGTTGGTGTCAACAAGATGG | *EF1a*-R | TGCCTTCAGCATTACCTTCC |
| D-*Th*-F | CCTCTGAGTGCGTATTGGTC | D-*Th*-R | GCTTATCATGGCAGCTTTCT |
| D-*Tβh*-F | GATTATCAGTTTCCCTTTCC | D-*Tβh*-R | TAGGTTGTCTCCTTTGTTGG |
| D-*Tdc*-F | GGCTATCAATAAGGCAGGTA | D-*Tdc*-R | CCTCCATCATCATTGTTACTG |
| D-*Tph*-F | TGCTTACTACTACACCGATTC | D-*Tph*-R | AATTGGATGTGGACAGCATT |
| D-*Hdc*-F | TCGTTCGGCATTAAGGGTCT | D-*Hdc*-R | CGATGGCGATTCTTGGTGAG |
| *GFP*-F | CGACGTAAACGGCCACAA | *GFP*-R | GGGTGTTCTGCTGGTAGTG |
| P-*Th*-F | TTGGACACCCTGAAAGACCG | P-*Th*-R | TAAGACGTTTGAGCGCCTGA |
| P*-Tβh*-F | CTTCGTGGCGTTCGTATTCT | P-*Tβh*-R | GCTGAGCGGTTCTTGGATGT |
| P-*Tdc*-F | GGAGGTGGAGTTATACAGAG | P-*Tdc*-R | CCGTTAAGAGTGACTTCAAT |
| P-*Tph*-F | AATTACACCACCATACATCC | P-*Tph*-R | GTCTTACGCTTCAAGTAGTT |
| P-*Hdc*-F | GGTGCTGTCCTTAATCCATA | P-*Hdc*-R | TAGTTGCCTATTGCTCTGAT |
| T7 sequence:TAATACGACTCACTATAGG | | | |

1. # Both authors contributed equally to this work.

   * Correspondence: Xin Yi (yixin423@126.com) and Guohua Zhong (guohuazhong@scau.edu.cn)

   Key Laboratory of Crop Integrated Pest Management in South China, Ministry of Agriculture, Key Laboratory of Natural Pesticide and Chemical Biology, Ministry of Education, South China Agricultural University, Guangzhou 510642, China. [↑](#footnote-ref-0)
2. [↑](#footnote-ref-1)
