# Supplementary material for: The modulatory effects of biogenic amines on male mating performance in Bactrocera dorsalis
Source: Front Physiol. 2022 Sep 6;13:1000547. doi: 10.3389/fphys.2022.1000547 (PMC9486026; doi:10.3389/fphys.2022.1000547)
Supplement: Supplementary file 2 [file Table2.DOCX]

**Supplementary Datasheet 1 Sequences used in this study.**

**>*Bactrocera dorsalis* tryptophan 5-hydroxylase 1**

ATGAGCGCTTCTGGTAAAAGTTTGCTGGGTCTTTGGCTCTATCGTAGTGGCGAGCAGGAGTGGGCTGTGAAACAGGGTAGCCCACTGCATACGTTACGCAAAGAACATCGCAGTCTAGATACCTCGAATTCCGAAAGTGGCAGTGAGAAAGGTAGTATTGATCTGAGCGATTCGAATCGCCAAAGTTCACCAGGAGATCGAGTGTCCATTATTTTCACCTTGAAAAATCAAGTTGGGAATTTAGCTAGAGCATTGCAAGTATTTCAAGAACTAGGCATTAATGTTCTACATTTGGAATTATCGCCTTTGGAGAAGGCAACCAATCAGGCTGACGTTCTCGTCGATGTGGAATGCGATGCTCGACGTTTGGATAAGGTACTGCAAATGTTAAACCGCGAGGTGCAGTCTGTAAATTACACCACCATACATCCGATGTCACGAACGCCATCGTTATCAGCCTGCTCCAGTTTTGACTTTGGAGAAATGGTCTGGTTTCCACGCAAAATATCCGATTTGGATAAAGCACAAAATGTGCTAATGTATGGCTCGGAACTCGATGCTGACCACCCAGGATTCAAAGATCCGGTCTATCGTAAGCGTCGCGAGACATTTTCTGCTATTGCTAACAACTACAAGAGTGGACATGCAATACCACGTGTGACCTACACACCTGAGGAGATAAAAACGTGGGGCACTGTCTTCCGTGAACTTCACCGCCTCTATATCAAGCATGCAGTGCCGGAGTATATGGAAAATTGGCCGGAATTGGTGAAGTACTGCGGTTACCGTGAGGATAATGTGCCTCAATTGGAGGATGTTAGTAACTACTTGAAGCGTAAGACGGGATTTCAGCTACGTCCAGTTGCTGGTTACTTATCGCCACGCGATTTTTTGTCTGGCTTGGCTTTTCGTGTTTTTCATTGTACGCAGTATATTCGACACTCATCGGATCCATTTTATACGCCCGAACCTGACTGCTGTCATGAGCTTCTGGGTCATATGCCATTACTAGCTAATCCAAGTTTTGCTCAGTTCTCTCAGGAAATTGGTTTGGCTTCGTTGGGAGCCGCGGATGCAGATATTGAAAAGTTGGCAACGTTATATTTCTTTACGGTGGAATTTGGTCTCTGCAAGCAACAGGATAACACTTTCAAAGTATACGGCGCTGGACTACTAAGCTCTATAGCTGAACTACAGCATGCCATAGCCGCTAAAGAGAAAATCAAAAAATTCGATCCGGAAGTTACGTGTCAAGAGGAATGCATCATAACCTCATATCAAAATGCTTACTACTACACCGATTCATTTGAGGAAGCCAAAGAGCAGATGAGAAATTTCGCTGATAGCATACAACGCCCCTTCGGTGTGCGCTACAATCCTTATACCCAAGAGGTCGAGGTGCTCTCGAATGCGCAGAAGATAACCGCTTTTGTGAGCGAGCTAAAGGGCGATTTGAGTCTGGTTTGTCAGGCAATGCGCAAGATTTCCGCAAACGATGAACAATTGGATGTGGACAGCATTGCAAATATGTTGCAAACAACATTGCATGTGCGCGGCGATCGAACGCCCGGCAGCTCGGTAAGTCCAGATAACTCAGATAACTCGCAGCACTCGGTTGGTGAATAG

**>*Bactrocera dorsalis* histidine decarboxylase**

ATGGATGTGAACGAATACCGACGACGGGGCAAAGAGATGGTGGACTATATCGCCGATTATTTGGAAAACATTCGTGACCGTCGCGTATTTCCCGATGTTAAGCCCGGTTATATGCGCGGTCTGTTGCCCGAGTTTGCGCCGATCGAAGGTGAAAATTGGGATGCCATATTCGCCGATGTGGAGCGCGTCATTATGCCTGGCATAACACATTGGCAAAGTCCACACATGCATGCCTACTTTCCAGCGTTGAATTCATTTCCTTCACTTCTCGGCGATATGTTAGCCGATGCGATTAACTGTTTAGGATTCACTTGGGCCAGTTCACCCGCTTGTACGGAACTCGAAGTGATTGTTATGAATTGGTTGGGCAAAATGATTGGCTTGCCAGATGATTTCTTGCATTTGCATAATAAAAGTCCAGGCGGTGGCGTCATACAGACGACGGCAAGTGAAGCTACGCTGGTGTGTCTGCTGGCGGGACGCACGCGAGCCATACAACGTTTCCACGAACGTCACCCCGGTTTTCAGGATGCGGAAATCAATGCGCGACTAGTTGCCTATTGCTCTGATCAGGCACACTCAAGCGTGGAGAAGGCAGCACTGATAGGGCTCGTGCGTATGCGTTTCATTGAGGCCGACGACAGCTTGGCGATGCGTGGCAAGGCGTTGCGCGAGGCCATCGAGGATGACATTAAGCAAGGACTCGTGCCGTTTTGGGTTTGCGCCACGTTGGGTACCACGGGCTCATGTTCCTTTGACAATTTGGAAGAGATTGGTATAGTTTGTCGCGATTTCAATATCTGGCTCCATGTCGATTGCGCTTACGCGGGCAGCGCTTTTATTTGCCCCGAATTCCGCACCTGGCTGCGGGGCATTGAGAAAGCAGATTCGATTGCCTTCAATCCATCTAAGTGGCTGATGGTACATTTTGACGCCACAGCATTATGGATTAAGGACAGCACCGCCGTACATCGCACATTCAATGTGGAACCACTTTACTTGCAGCATGAAAATTCTGGCGTCTCCATTGACTACATGCATTGGCAGATACCGTTAAGTAGACGCTTTCGCGCTTTGAAAGTTTTCTTTGTATTGCGTTCGTTCGGCATTAAGGGTCTGCAAAAGCATATACGCGAAGGCGTACGTTTGGCGCAAAAGTTCGAAGCGCTCGTGTTGGCCGACCACCGTTTCGAGATACCGGCTAAACGGCACTTGGGTAAGGCTATATTTGATGCCGACGAATTTTTGGCCAAAACCTATGCTGGCGTACGTATTGCTCACCAAGAATCGCCATCGATGCGGCGACGTGTGCGCGGTATACTCATGTCTGGCAAACAGTTTTCACTAGATTCCCATATGGATGTGGTCGTGCAGAGCTCGTTCGATTCGGGTACGAATAATTCAAGCACGGAGGCAAATGGCAGCACAACACCAGTAAAAAAGAACAAAAATCCAAGCTCGATATGCGAAGATAGTGAAGAGTCCGCCGAAGATTATCCAACGGTCAAGCGAACCGCAACGTCGCCAAACTATTCAACGATGAAGATAGCGGCTGAATATCTGAGCCAAAACTCGATCTCCGCTACAACGAACCTCGCATCGCCACGTCGCAAAAGCACCGCCACGCAAACACCGCAACATCACTACAAGCCGTTGCATGCCACCTTAGATGAAGTCTCCGATGAGACGTCCGAGGACTTGCTCTCGGAGAAAAGTGTAATGGACACCGCGAATGCGCAGCTAGTGGCCGCTAGTTGCAAGCTACTTCTAGCAAATACACAACCAACTCTTGCAGCAACCTCCTCCACGGCGCCTGCCTCGCTGCGCACAACGCCATCACCGCCTCTGCCAGTGCCGCAACAGCAGCGCGCAGGCAATAATAATAATTTCACACAACAAATGCAAGCGATCAGTAATGCGGCAAGCGCTTCAACGCGCACCATCATGCGCGCCAACACCATGCAAGAGGTCTATCATGGTAAGCGTTACCTCGACACAGGTGTGGCTGTGGGTCCAGCCAACAATTACTTCTCCAACACCATAATCAATGCGAACACGCCGAGCGGCGGCACCACACCGACAACACCGAGCGCCGAAACACCGGACGCTGAATCGCAATGGAGCGCGCTATTTCACAGTTTCGATACCGCGCCGGCGCCCACCGTGCGCGGTGTGAGCTATTTGAGCGGTCGTCTACTCAATATGGATAGCGTTTCGGCGCGCTCCACTTCAACGCTGCCGAGTATTGACGAAAGCGCTTCCGAATGCATGTCCATCTCATCGACGTCCACACCGGCGGACACATTCAGTATGCCTGCGCAGCTGTTGTCGAACCGACACAATGTAACACGCTTTTACAGCGCGCCAAGCGCGCGTCCGGCTGCGCCGTCAGCAACAATGCCAAGCACGTTGGCGTCATTTTCGTTAAACGCCATGACACTCTCCGAGGATGAGACGGACGCGGATGCCTGGCAGGTGGCGCGTTGCGGCAGTTCGGAGGATTACTCGATCACTTCATCGGGTGCACAATCGCGTTCTTCGTCGTTAGATCTGGTTTGA

**>*Bactrocera dorsalis* tyrosine decarboxylase**

ATGGATAGTCAAGCATTTCGTAAGCATGGCACAGAGATGGTGGAATATATATGCAAATATTTGGACACCCTGAAAGACCGGCGTGTCACACCTAGCGTCGAACCCGGCTATTTAAGACATTTAATACCATCTGAGGCTCCATTCGAACCGGAGGAATGGAGTAAGATAATGCAAGATGTGGAAGATAAAATAATGCCTGGTGTCACGCATTGGCAGCATCCACGATTTCATGCCTACTTTCCGGCAGGAAATGCATTCGAATCGATTTTGGGTGATATGCTGGGGGATGGTATCGGTTGCATTGGTTTTTCCTGGGCCGCAAGTCCCGCATGTACGGAGCTGGAAACAATTGTGTTGGATTGGCTGGGAAAAGCAATCGGTTTACCTGACCATTTTTTAGCTTTAAAGGAGGGCAGCACAGGAGGTGGAGTTATACAGAGTTCCGCCTCTGAGTGCGTATTGGTCAGCATGTTGGCCGCGCGCGCTCAGGCGCTCAAACGTCTTATGGCTCAACATCCATTTACCGAGGAAAGTCACTTACTTTCCAAGTTGATTGCCTATTGCTCCAAAGAAGCACACAGTTGTGTTGAGAAAGCTGCCATGATAAGCTTTGTAAAACTGCGAATTTTGGAGCCAGATGAAAATGCTAGCTTGCGCGGGCGCACCGTGAGTGAGGCAATGGAGGAGGACGAATTGCAAGGTTTGGTGCCATTTTTCGTTTCCACCACCCTTGGCACTACCGGCTCCTGCGCCTTTGATAACTTGGAGGAAATTGGCAAGGAATTGAAAAAGTTTCAGTGTGTTTGGTTGCATGTGGATGCTGCCTATGCCGGCAACTCTTTCATCTGTCCCGAATTGAAGTCACTCTTAACGGGTATCGAATACGCTGATTCATTCAATACGAATCCCAATAAATGGCTATTAACAAATTTCGACTGTTCAACATTCTGGGTCCGCGATCGAATCCGCTTGACATCAGCACTTGTCGTGGATCCATTGTATCTGAAGCATTGCTATGATGATGCAGCAATTGACTACCGTCATTGGGGAGTTCCGCTCAGTCGACGTTTTCGCTCGTTGAAATTGTGGTTTGTGTTGCGTTCCTACGGAATTTCCGGATTGCAGAACTACATACGACGTCACATCGAATTGGCCAAACGTTTCGAACAGCATGTGCTTAAAGATAAACGCTTCGAGATTTGTAATCAAGTCAAGCTTGGATTGGTTTGTTTCCGATTGAAAGGTAACGACAAGCTAAATGAGAAGCTACTCAGCGCCATTAATGAATCAGGTAAACTGCATATGGTTCCTGCTAGTGTCAACGATAAATACATTATACGATTTTGTGCGGTGGCACAAAATGCCTGTGAAGACGACATCGATTATGCCTGGGAAACCATAGTTGATTTTGCTAACGAATTACTTGAAAAGGAACAACACGATGAACTAACAGAGATAATCAATCGAAAGAAGGAAGACACATTGGCTAAGAAACGCTCATTCTTCGTACGAATGGTCAGCGATCCGAAAATCTATAACCCGGCTATCAATAAGGCAGGTACGCCAAATATGTCGTTAGATGTTACATCGCCGGTAGTGGGACACTCTTCAAATCCTATTATAAGAACACAAACATCGATGGATCGGAATTCGTGGATTTCCTGGCCCTTAGCGTTTCTCTTCAACAGTAACAATGATGATGGAGGGCAGAAGAGTAATGTTTCATTGCGTTTTCGTCATTTAGACACAAATGTACGAGCAATTTCGTCGAGACGCAATTCCGGTGCCACATCTTCGCCATCACCTGACAATGAGTTGGGCTCTATTAATCTGAAGAAGTCACCAATAAAACCAACATCACCATCGCCACGAAAAGGCGGCCTCTCACTAACCACAACTAGCAATCACTCCTAA

**>*Bactrocera dorsalis* tyrosine 3-monooxygenase**

ATGGATAGTCAAGCATTTCGTAAGCATGGCACAGAGATGGTGGAATATATATGCAAATATTTGGACACCCTGAAAGACCGGCGTGTCACACCTAGCGTCGAACCCGGCTATTTAAGACATTTAATACCATCTGAGGCTCCATTCGAACCGGAGGAATGGAGTAAGATAATGCAAGATGTGGAAGATAAAATAATGCCTGGTGTCACGCATTGGCAGCATCCACGATTTCATGCCTACTTTCCGGCAGGAAATGCATTCGAATCGATTTTGGGTGATATGCTGGGGGATGGTATCGGTTGCATTGGTTTTTCCTGGGCCGCAAGTCCCGCATGTACGGAGCTGGAAACAATTGTGTTGGATTGGCTGGGAAAAGCAATCGGTTTACCTGACCATTTTTTAGCTTTAAAGGAGGGCAGCACAGGAGGTGGAGTTATACAGAGTTCCGCCTCTGAGTGCGTATTGGTCAGCATGTTGGCCGCGCGCGCTCAGGCGCTCAAACGTCTTATGGCTCAACATCCATTTACCGAGGAAAGTCACTTACTTTCCAAGTTGATTGCCTATTGCTCCAAAGAAGCACACAGTTGTGTTGAGAAAGCTGCCATGATAAGCTTTGTAAAACTGCGAATTTTGGAGCCAGATGAAAATGCTAGCTTGCGCGGGCGCACCGTGAGTGAGGCAATGGAGGAGGACGAATTGCAAGGTTTGGTGCCATTTTTCGTTTCCACCACCCTTGGCACTACCGGCTCCTGCGCCTTTGATAACTTGGAGGAAATTGGCAAGGAATTGAAAAAGTTTCAGTGTGTTTGGTTGCATGTGGATGCTGCCTATGCCGGCAACTCTTTCATCTGTCCCGAATTGAAGTCACTCTTAACGGGTATCGAATACGCTGATTCATTCAATACGAATCCCAATAAATGGCTATTAACAAATTTCGACTGTTCAACATTCTGGGTCCGCGATCGAATCCGCTTGACATCAGCACTTGTCGTGGATCCATTGTATCTGAAGCATTGCTATGATGATGCAGCAATTGACTACCGTCATTGGGGAGTTCCGCTCAGTCGACGTTTTCGCTCGTTGAAATTGTGGTTTGTGTTGCGTTCCTACGGAATTTCCGGATTGCAGAACTACATACGACGTCACATCGAATTGGCCAAACGTTTCGAACAGCATGTGCTTAAAGATAAACGCTTCGAGATTTGTAATCAAGTCAAGCTTGGATTGGTTTGTTTCCGATTGAAAGGTAACGACAAGCTAAATGAGAAGCTACTCAGCGCCATTAATGAATCAGGTAAACTGCATATGGTTCCTGCTAGTGTCAACGATAAATACATTATACGATTTTGTGCGGTGGCACAAAATGCCTGTGAAGACGACATCGATTATGCCTGGGAAACCATAGTTGATTTTGCTAACGAATTACTTGAAAAGGAACAACACGATGAACTAACAGAGATAATCAATCGAAAGAAGGAAGACACATTGGCTAAGAAACGCTCATTCTTCGTACGAATGGTCAGCGATCCGAAAATCTATAACCCGGCTATCAATAAGGCAGGTACGCCAAATATGTCGTTAGATGTTACATCGCCGGTAGTGGGACACTCTTCAAATCCTATTATAAGAACACAAACATCGATGGATCGGAATTCGTGGATTTCCTGGCCCTTAGCGTTTCTCTTCAACAGTAACAATGATGATGGAGGGCAGAAGAGTAATGTTTCATTGCGTTTTCGTCATTTAGACACAAATGTACGAGCAATTTCGTCGAGACGCAATTCCGGTGCCACATCTTCGCCATCACCTGACAATGAGTTGGGCTCTATTAATCTGAAGAAGTCACCAATAAAACCAACATCACCATCGCCACGAAAAGGCGGCCTCTCACTAACCACAACTAGCAATCACTCCTAA

**>*Bactrocera dorsalis* tyramine beta-hydroxylase**

ATGGCTGTAAAAGGCAAGCTATCAAGCGCTCACGGCAGTTGGACGTCCTCCAGAGTCGGCACCACGTCCTACATATCGCGCATGGCTTGTTTCGCCTTTTGCCTGCTCTCCGCTCTGCCGCTGCCGTTCTTCACAACAGCTAATCGTTTATCTGACACCAAACTCCATGAAGTATATCTAGACAACTCCGAAATAAAGCTAAGTTGGATGGTGGATTGGTATAAACAGGAGGTGCTCTTTCATCTACAAAATGCCTTTAACGAAAATCACCGCTGGTTCTATTTGGGCTTTTCGAAGCGTGGCGATGTGGGCGACGCTGACATCTGTTTCTTTGAGAATCAAAATGGATTTTTCAACATTGTCACCGACACTTATACCAGTCCAGATGGTAAATTTGTGCAAAAAGACTATCAACAAGATTGCGAACTTTTCAAAATGGATGAATACACGTTGGCATTTAAGCGAAAATTCGACACCTGCGATCCGCTGGACTTGAGGATGCACGAGGGTACAATGTACATTATGTGGGCCAGAGGTGAAAGTCGCTTGGCACTGGAAGATTATCAGTTTCCCTTTCCTAATATTTCCTCACATGAATCTGGTATTAAGATGATGCAATTACTTCGCGCAGATAAAATTCTCATACCAGAAACCAATTTGAAAACAATTCAAGTCACACTCGAAAATGTGACCGTACCAACAAAGGAGACAACCTACTGGTGTCACATACAAAAACTGGACGAATCATTTGCGACGAAACAGCATATAGTGCAATTCGAGCCACTCATTACCTCTCCAGATTTGGTGCATCATCTGGAAGTTTTTCACTGCGAAACCGATCCACAAGTAGAAATTCCATTATACAATGGAGACTGTGAGAAAATGCCGGCAGAAGCTAAAGTGTGCTCCAAAGTGATATCACTGTGGGCTATGGGCGCTAGCACGTTCACGTATCCACCAGAAACTGGCTTACCAGTAGGAGGCAAGGACTATAATCCATTCGTGCGCCTAGAAGTGCATTTTAACAATCCCGAATTGAAAGCAGGTCGCATTGATAGCTCTGGCATGAGAATCAAGTTGGCTTCGAAGCTAAGACAGTTCGATGCGGGCGTAATGGAATTGGGCTTAGAGTACACCGATAAGATGGCTATACCACCCGGTCAAGTGGGCTTCCCGCTAAGTGGTTATTGTATCGCCGAATGTACTGAAGTGGCCCTTCCGTCCAGTGGTATTATCATATTCGGTTCTCAACTGCATACACATCTTCGTGGCGTTCGTATTCTAACACGTCATTTTCGCGATAACGAGGAACTGCGCGAAGTCAATCGAGATGACTATTACTCGCATCATTTTCAAGAAATGCGCAACCTGCACTATAAACCTCATGTGCTACCGGGTGATGCTTTGGTCACAACTTGCTATTATAATACGCTGGGCTATGAAAATGCAACACTGGGCGGTTTTTCAATCAGCGACGAGATGTGTGTGAACTATATTCATTATTATCCGGCTACCAAGTTGGAGGTTTGCAAAAGTTCTGTCTCCGAAGATACGCTGGAAAATTATTTCATTTATATGAAAAAGAAAGAACATCAACGCGGCATAAGGCTAAGTGGAGCACGTTCGAAGAATTATCGCAGCATTAAATGGACGGAACCACGTGTGGATCAACTTTACACCATGTACATCCAAGAACCGCTCAGCATGCAGTGCAACAAATCGGATGGTTATCGCTTCGAGGGCTACAATTGGGAAGGAGCACCAGTGACGCCAGTGGCAATCAAAATACCCATGCACAGCAAACTATGTCCCAACTACAACCCGTTATGGTTGAAACCGCTGGAAAAGGGTGCGTGCGACTTACTGGGCGAATGTATCTATTAA
